# Informing Comprehensive HIV Prevention: A Situational Analysis of the HIV Prevention and Care Context, North West Province South Africa

**DOI:** 10.1371/journal.pone.0102904

**Published:** 2014-07-16

**Authors:** Sheri A. Lippman, Sarah Treves-Kagan, Jennifer M. Gilvydis, Evasen Naidoo, Gertrude Khumalo-Sakutukwa, Lynae Darbes, Elsie Raphela, Lebogang Ntswane, Scott Barnhart

**Affiliations:** 1 University of California San Francisco, Center for AIDS Prevention Studies, San Francisco, California, United States of America; 2 University of Washington, International Training and Education Center for Health (ITECH) – South Africa, Pretoria, South Africa; 3 University of Washington, International Training and Education Center for Health, Seattle, Washington, United States of America; Rollins School of Public Health, Emory University, United States of America

## Abstract

**Objective:**

Building a successful combination prevention program requires understanding the community’s local epidemiological profile, the social community norms that shape vulnerability to HIV and access to care, and the available community resources. We carried out a **situational analysis** in order to shape a comprehensive HIV prevention program that address local barriers to care at multiple contextual levels in the North West Province of South Africa.

**Method:**

The situational analysis was conducted in two sub-districts in 2012 and guided by an adaptation of WHO’s Strategic Approach, a predominantly qualitative method, including observation of service delivery points and in-depth interviews and focus groups with local leaders, providers, and community members, in order to recommend context-specific HIV prevention strategies. Analysis began during fieldwork with nightly discussions of findings and continued with coding original textual data from the fieldwork notebooks and a select number of recorded interviews.

**Results:**

We conducted over 200 individual and group interviews and gleaned four principal social barriers to HIV prevention and care, including: HIV fatalism, traditional gender norms, HIV-related stigma, and challenges with communication around HIV, all of which fuel the HIV epidemic. At the different levels of response needed to stem the epidemic, we found evidence of national policies and programs that are mitigating the social risk factors but little community-based responses that address social risk factors to HIV.

**Conclusions:**

Understanding social and structural barriers to care helped shape our comprehensive HIV prevention program, which address the four ‘themes’ identified into each component of the program. Activities are underway to engage communities, offer community-based testing in high transmission areas, community stigma reduction, and a positive health, dignity and prevention program for stigma reduction and improve communication skills. The situational analysis process successfully shaped key programmatic decisions and cultivated a deeper collaboration with local stakeholders to support program implementation.

## Introduction

South Africa’s epidemic remains the world’s largest with an estimated 6.4 million people living with HIV [1], an adult HIV prevalence of 17.8% in persons aged 15 to 49 [2] and an estimated 469,000 new HIV infections occurring annually [1]. South Africa also has the third highest TB burden in the world and 63% of all TB cases in South Africa are among people who are HIV-positive [3]. In response, the South African government has undertaken mass HIV-testing campaigns and changed its national policy in 2011 to provide ART for people with CD4 counts of 350 cells/µL or less from the previous threshold of 200 cells/µL [4] as well as for HIV-positive women who are pregnant and breastfeeding and persons with a WHO clinical stage 3 or 4 irrespective of CD4. Despite these efforts, HIV testing and treatment programs still fall short of levels needed to have a significant impact on the epidemic. National data from 2012 indicated that 65% of the population had ever tested for HIV; however, only 37.8% of HIV-positive men and 55% of HIV positive women were aware of their HIV status [1]. Additionally, while South Africa has the largest antiretroviral program in the world [5], it is estimated that only 52% of patients eligible for ART in South Africa are currently on treatment [6]. As of 2011, TB cure rates were still only estimated at 77% nationally [3].

With the HIV and TB co-epidemics still rampant despite years of prevention efforts, the scientific and programmatic communities have embraced a comprehensive prevention approach. Comprehensive prevention is predicated on the idea that no single intervention is efficacious enough to bring an HIV epidemic under control on its own, instead, integrated biomedical, behavioral, and structural strategies should be utilized as they are more potent in combination [7], [8]. Intervention components offered together increase both the likelihood of meeting the needs of a diverse population and the potential for improved effectiveness due to synergy from complimentary approaches (e.g. enhanced counseling may increase the effectiveness of pre-exposure prophylaxis (PrEP); community engagement can increase demand for HIV testing). As the set of biomedical, behavioral, and structural tools available in the prevention toolbox expands, the question remains: What combination of programming is most effective for each community given their specific barriers to care, resources, determinants of health, and cultural contexts?

Building the ideal combination of prevention tools with appropriate thematic focus and messaging depends on an understanding of the community’s local epidemiological profile, the religious and social community norms that shape vulnerability to HIV and access to care, and the available community resources and local preferences [9]. For example, gender inequity, poverty, and migration are often key social factors that shape the HIV epidemic, but these phenomena do not occur consistently across countries and populations; therefore, programming should address context-specific risks that result from the social environment [10]. Additionally, community norms regarding prevention tools, (e.g., condoms, HIV testing) as well as sexual behaviors and partnership patterns in a community (concurrency, sex work, early marriage) shape local HIV epidemics [11]. The success of prevention programs hinges on elucidating and understanding how specific social factors and community norms interact within the local context and then identifying the appropriate tools and approaches to address these at multiple levels to improve health outcomes [12].

In preparation for design and implementation of a US Centers for Disease Control and Prevention (CDC) and South African Government supported comprehensive prevention program, our team undertook a situational analysis in two districts in North West Province, South Africa, in order to ensure that programming was responsive to the local context. The situational analysis was undertaken to characterize the communities’ needs, existing resources, and cultural and structural barriers to health care. In this manuscript, we describe the situational analysis approach, present qualitative findings from our fieldwork, discuss the results in the context of South Africa and prevention programming at multiple contextual levels, and conclude by discussing how these findings have been translated into comprehensive strategies tailored to the context. While the overall situational analysis sought a broad characterization of the epidemiological profile, service delivery needs, and programming gaps, this paper focuses on the characterization of the social and structural factors that act as barriers to healthy behaviors and service uptake. We present the results organized by the four social factors or ‘themes’ identified as primary barriers to HIV prevention.

## Methods

### Assessment Approach

A situational analysis is the process of building an expansive understanding of the epidemic in context, including review of available information, exploring new information, and triangulating varied data sources [13]. The process consists of building partnerships and broad opportunities for input from local stakeholders to ensure construction of a complete picture of the HIV/AIDS epidemic in a given community, its causes, and potential responses. Based on PEPFAR’s and UNAID’s “Know your epidemic, know your response” model [7], our situational analysis focused on characterizing three components: the epidemic profile in the area, the socio-cultural and service delivery contexts, and successful programs and best practices, including community resources that can be leveraged for program implementation and synergies with local government [7]. We also sought to identify target communities, municipalities and venues for prevention activities and to plan and facilitate integration of program activities into the district health services. Finally, during the assessment stage we identified potential members for community working groups (CWGs) as a way of effectively engaging the communities in prevention planning.

Data collection was guided by an adaptation of the assessment stage of the WHO’s three-stage strategic approach [14], which is primarily qualitative and permits ample flexibility in the field. The WHO strategic approach provides a framework to explore both the health system and the socio-cultural and political environment shaping community needs in order to inform decision-making [14]–[17]. Our assessment had four basic steps: 1) *laying the foundation,* which included developing partnerships with the local government, defining objectives and forming a core team; 2*) preparing for fieldwork,* which included identifying research sites, holding stakeholder’s workshops in each site, developing data collection instruments, and training the field team; 3) *conducting fieldwork,* including in-depth interviews, focus groups, and facility and NGO assessments; and 4) *reporting back,* including integrating findings into programmatic work, writing reports and eliciting feedback, and holding a dissemination workshop. More detail on processes is outlined elsewhere [14], [18].

### Study Sites

The assessment was undertaken in two sub-districts in the North West Province of South Africa, the Province with the fourth highest HIV prevalence in the country (30.2% of pregnant women testing HIV-positive according to the 2011 National Antenatal Sentinel HIV and Syphilis Prevalence Survey [19]). The sub-districts for study, Moses Kotane and Naledi, were identified by the district health offices at the Department of Health (DOH) as two areas prioritized for program implementation. Both sites are characterized by poverty, unemployment and low literacy levels (see table 1). Moses Kotane sub-district in Bojanala Platinum (BP) district is spread over a vast, rural geography and is characterized by heavy travel due to the proximity to two national highways, the mining industry, and Sun City, a large casino and tourist attraction. Naledi is the hub of government, NGO and economic activities in Dr. Ruth Segomotsi Mompati district (Dr. RSM), and is the agricultural hub of the district. The area is more racially diverse than Bojanala Platinum, although racial communities are geographically and socially separated.

**Table 1:** Characteristics of Study Sites.

	Moses Kotane, BP	Naledi, RSM
Total Population†	242,554	66, 781
Black African	238,516 (98.34%)	49,423 (74.01%)
Coloured	620 (0.26%)	9,827 (14.72%)
Indian or Asian	1,200 (0.49%)	748 (1.12%)
White	1,829 (0.75%)	6,352 (9.51%)
Other	389 (0.16%)	429 (0.64%)
Unemployment rate*	25.7%	28%
% of households earning ≤R1600 *(approx equivalent to $225)* *	76%	83%
% not receiving any schooling†	7.5%	13.6%
Principle Industries	Mining, tourism	Farming, government
**District** antenatal HIV prevalence**	33.9%	20.5%*
Number of hospitals	1	1
Number of Community Health Centres	3	2
Number of Primary Health Clinics	47	3
Number of mobiles	3	4

† Stats SA 2011 Community Survey [74].

*StatsSA. 2007 Community Survey - Labor Force [75].

**Department of Health.2011. The National Antenatal Sentinel HIV and Syphilis Prevalence Survey [19].

### Procedures

Prior to field work, stakeholder’s workshops were held in each sub-district. Stakeholders included district and sub-district health officials, municipal and traditional leadership, NGO representatives, PEPFAR partner organizations, health providers, and traditional healers. At the workshops, a background paper outlining existing epidemic and service delivery data and gaps in knowledge was presented and discussed. The stakeholders then assisted the team in identifying priority themes and participants and venues of interest to visit during fieldwork through a community mapping exercise. These priorities shaped the data collection in each community, where a team of 9–11 trained researchers spent one week visiting health and social service locations, NGOs, and areas where vulnerable and mobile populations congregate, such as taxi ranks, taverns, places of employment such as mines and farms, and informal settlements.

The fieldwork included in-depth-interviews, focus groups, and service inventories and observations. Participants were selected through a combination of convenience and purposeful sampling – based on stakeholder recommendations and an attempt to obtain a cross-section of age, gender, and socio-economic profiles. The interview and visit schedule was flexible and modified as necessary as fieldwork progressed to ensure broad inclusion of all key groups and informants and to follow leads as they arose. Semi-structured interview and focus group guides included questions regarding community characteristics, mobility, employment, health challenges, TB and HIV-related services, stigma related to HIV or TB, sexual partnerships, and suggestions for health programming in the area. Health facility and NGO assessments were conducted to document local resources and gaps in services. Written informed consent preceded all interviews; consent and interviews were conducted according to the language of choice: English or Setswana. Researchers took detailed field notes of each interaction and recorded interviews when participants consented to be recorded. All procedures were approved by the Committee for Human Research at the University of California, San Francisco (UCSF) and the Human Sciences Research Council (HSRC) Research Ethics Committee in South Africa. The Human Subjects Division at University of Washington and CDC’s Center for Global Health Human Research Protection reviewed and approved the study prior to implementation.

### Data Analysis

A unique aspect of this approach is the iterative analytic process undertaken in the field [16]. During the fieldwork, researchers met at the end of each day to summarize and discuss findings, pinpoint unanswered questions, offer new leads, and prioritize subsequent interviews. These meetings were documented around three themes: the local HIV/TB epidemic; service delivery, including available health facility and NGO resources, barriers to care, gaps in services; and social-cultural influences on health. By the end of the week, the research team had agreed on the principal themes that were emerging from the data, on key venues and areas to focus prevention efforts, and on key questions that required further investigation.

Upon completion of data collection, analysis consisted of reviewing meeting notes, reading and coding original textual data from the fieldwork notebooks, and transcribing and coding of a select number of recorded interviews. Interviews were selected for transcription purposefully to ensure inclusion of diverse informants (i.e. program officials, providers, clinic patients, community members) and key information from informants where field notes were incomplete or language of interview made it difficult to cull important themes from mixed language notes. Overall 31 interviews were transcribed and entered into Altas.ti for coding and further analysis; interviews in Setswana were translated and transcribed by a transcription service and reviewed by bilingual field staff for accuracy. Codes included community characteristics (mobility, poverty, risk groups); health behaviors and norms (awareness and concern, condom use, testing, disclosure, treatment); health care and barriers to accessing care (service availability, quality); and social structural factors (stigma, gender, violence). Based on review of the deductively coded data, the authors developed inductive codes to flag data on themes that emerged within or across the topics in Atlas.ti. Code reports were generated and reviewed; data notebooks were re-read after code reports were produced to cross-check findings. Authors discussed and clarified the main ideas that emerged within the different code reports and explored relationships within the data among themes [20], [21]. As themes were highly consistent across sites, data on the two sub-districts are presented in aggregate.

We present the major social contributors to HIV vulnerability noted in the assessment: HIV fatalism, traditional gender norms, HIV-related stigma, and challenges with communication around sex and HIV, all of which exist and transpire within the larger context of poverty and lack of employment opportunities. We first present these principal social contextual contributors to HIV vulnerability as captured in the data and then explore current and potential responses that address these social factors at multiple levels. This multi-level frame has shaped the programming that addresses these contextual barriers to care at the community, organizational, clinic, and policy levels.

## Results

Between the two sites, 334 key informants, providers, and community members consented to participate in 203 in-depth interviews and focus groups during approximately 15 days of fieldwork in March and May, 2012. Key Informants included provincial, district, and sub-district officials in government and within the health sector, as well as traditional leadership (DiKgosi or chiefs) and program directors in non-governmental social or community programs. Providers included physicians, nurses, pharmacists, auxiliary nurses, HIV counselors, and lay health workers, mostly in the public sector. In addition, 13 health facilities and 7 NGO assessments were conducted (Table 2).

**Table 2:** Number of participants in Interviews, Focus Groups and Assessments, by place and type.

Information Source	Number of Participants		
	Moses Kotane	Naledi	TOTAL
Provider Interview	26	36	62
Key Informant Interview	20	34	54
Community Member Interview	28	53	81
Community Member Focus Group	74	63	137
Health Facility Assessment	7	6	13
NGO Assessment	2	5	7

### Service delivery context

While health care is free in government health facilities, community members in remote areas reported significant challenges accessing care. The principal barrier was poor roads, some of which become impassable during the rainy season, followed by the inability to pay for transport to reach the facilities, and, for some, a preference to use more distant facilities for purposes of confidentiality or a preference to avoid known providers and community members. Moses Kotane has a large number of health facilities distributed across the sub-district. Conversely, Naledi, has one quarter of the population in a much smaller geographic space and is the hub of all government services in Dr. RSM. As such, the local health department is able to coordinate with other government agencies and NGOs in the area to reach more distant communities. However, small pockets of the population live on farms and experience more difficulty in accessing services in Naledi, where communities also remain largely segregated by race (e.g. White, Black, Colored, Indian).

### HIV fatalism: a context of elevated HIV prevalence

Community members were generally well informed about the mechanisms of HIV infection and noted that condoms, HIV counseling and testing (HCT), and HIV and TB treatment were readily available. Several community member, provider, and key informant participants noted that the rates of HIV-related deaths have declined with the roll out of ART. However, the broad availability of information about HIV transmission in the area and general knowledge about transmission did not seem to translate into protective behaviors. Informants knew where to test for HIV and how to prevent infection, however very few informants reported regular testing and consistent condom use. Most participants maintained that condom use is not mainstream practice and preferred not to seek HIV testing, particularly men. Instead of reporting widespread protective measures, people reported a sense of complacency about HIV, in that eventual infection was inevitable.

*“Well, at first they were [concerned about HIV] but now it’s treated like just any other illness, you know, whether you get it or you don’t get it. You know there’s a saying that’s going around that all of us are going to die of it, so there’s a bit of a complacency that comes with that.”* [key informant]

*“People no longer care about HIV; they take it as any other disease. They say: they will die anyway.”* [provider]

This degree of HIV fatalism accompanied many of the interviews, as did reports of knowing many community members who had died (or suspected to have died) from HIV. There was a sense that people *“take their own life for granted”*. Others said that people lose hope because they have lost loved ones and still others felt that youth have ‘given up’ on prevention. One key informant said:

*“It seems to be we are fighting a losing battle. People continue being infected. I don’t see it coming to an end.”* [key informant]

Despite the fact that almost all participants identified HIV as a challenge in their community, there was also very little evidence of any community-driven response around resolving or mitigating the burden and further spread of HIV. There were rare instances of community groups, often limited to a handful of Home Based Care (HBC) workers, who appeared to be active in HIV/AIDS related concerns. Otherwise, HIV seemed to rank low on the priority list of numerous community hardships, including reported high rates of violence, alcohol use, unemployment, and poverty – all simultaneously crippling the community’s social fabric.

### Traditional Gender Norms, Violence, and Economics

Despite South Africa having one of the most gender-equitable constitutions in the world, the data suggest that significant separation of gender roles and gender inequity remains the norm. Gender inequity and adherence to traditional gender norms–or society’s conforming to definitions of masculinity that emphasize adventure, risk-taking, seeking multiple sexual partners, and adversarial and inequitable attitudes towards women [22]–[26] –were major themes throughout the interviews in both sites. One key informant said:

*“It’s gender disparities that we still experience in our communities. Where males still feel we are living a patriarchal society”* [key informant]

Gender inequity seemed to have significant impact on HIV in the area including women relating reduced power to negotiate condom use, expectations that men should and would have concurrent partnerships, and consistent reports of blaming of women for HIV transmission in men’s narratives of the HIV epidemic. Providers consistently said that men rarely get tested for HIV and are generally reluctant to utilize health services – they prefer to send their female partners for HCT or to test only when signs and symptoms need attending to. The result is both extreme delays in care for men and the burden (and blame) of health care responsibility placed on women, though they have little power to negotiate condom use to keep themselves healthy.

*“Some women prefer not to tell their husbands because the husband will say that, ‘I don’t have the disease. You have it.’ Remember, the men have not tested. The women tested positive and tells the husband. And [the husband says], ‘uh-uh, it’s you.’” [key informant]*

In addition, participants reported high rates of intimate partner violence and rape. As with HIV generally, there seemed to be little community response to violence and a degree of normalization of violence in the community, particularly violence again women. People reported stories of violence in matter-of-fact tones, giving the impression that these occurrences are commonplace and have become so common as to be non-controversial.

*“Boys will lock girlfriends in their houses. It will get reported to the police but the boys are back [in the community] the next day. The boys say the girls deserve it.”* [community member, female]

A significant and iterative link between HIV, gender norms, and economic dependence also emerged in the data. Informants reported significant stigma and silence around sex, especially for women, as discussions of sex and HIV are associated with ‘promiscuity’. At the same time, the high rates of unemployment and poverty created an environment where the community implicitly condoned sex as a tool for attaining basic needs. In many interviews, informants were asked about formal sex work, which most respondents associate with a structured establishment and said was not common. However, if asked about sexual partnerships in the context of economic exchange or security, most informants indicated that this was quite commonplace and diverse – ranging from pursuit of food and shelter to seeking entertainment and clothing. The most common narrative was that of young women seeking stability (food, housing) for themselves and young children, particularly from older men, which further perpetuates uneven power dynamics and potentially fuels HIV transmission. One female key informant from Moses Kotane said:

*“There’s no sex work but I think there’s a lot of … girls who don’t mind having sex with guys just to get money… The community does know about it but, you know, if you’re living with parents and you’re coming home with a bag of mealy meal and some meat - they don’t ask you where you get the money for that. In a way, they are kind of endorsing it.”* [key informant]

Informants’ narrative around sex work indicated a tension around social norms that women should not be sexually active outside of marriage (which is also endorsed by the large presence of evangelical churches), and recognition that women have extremely limited employment options in such a low resource setting. This social dynamic served to perpetuate silence, encourage shame and stigma around sex and HIV, and left women with few alternative sources of support or means to openly discuss their challenges with regard to HIV and preventing HIV infection.

### HIV stigma in the community and clinic

The creation of national anti-discrimination laws and the implementation of aggressive, national HCT and treatment campaigns – which included anti-discrimination messaging and a focus on healthy living with HIV [27] – represent increasingly progressive policies and government acknowledgement regarding the need to address stigma. However, stigma remains ubiquitous in the community and is still enacted frequently in the health sector, though there are some signs that stigma is dissipating. Informants reported that when people learn they are HIV-positive they hide it from their families and from the community and that disclosure leads to discrimination, ostracism and violence. A community member reported her perception of what happens if someone discloses their HIV status to family:

*“Now [the family member] thinks ‘that person doesn’t have to eat with my spoon, doesn’t have to wash my dishes, doesn’t have to sleep with us, he has to sleep alone.’ That is making a stigma; that’s why I think most of the people they died because of that… [they think] ‘I’m HIV positive, which means I don’t have anything left to do. Only one thing that I must do, I must die.’ And the person ends up dying.”* [community focus group, female]

Even providers, who model behaviors for the community, were reported to avoid disclosure. One provider stated:

*“Staff members do not disclose their status due to fear of rejection and stigma by others…”* [provider]

Most participants also recognized stigma as a major barrier to accessing HIV testing, care, and treatment. Community members and providers reported that people were hesitant to seek care and risk becoming “known” as HIV-positive to others, leading to late diagnosis and poor prognosis for treatment. Community informants repeatedly mentioned not accessing services because health care workers would judge them and recounted stories of health facility staff exhibiting discrimination towards HIV-positive patients or failing to keep HIV status confidential. For example, one key informant described a breach in confidentiality by a local health provider:

*“Recently someone (health care provider)…. was drunk…. and said that our village is full of people who are HIV-positive and that s/he could even expose them by their names. This person even mentioned another family by name. As a result many are scared to get their treatment… because they do not want their names spread all over the place.”* [key informant]

At the same time many providers as well as some key informants and community members said that HIV-related stigma was on the decline. Particularly, a number of providers and key informants used language describing HIV as a chronic disease, comparing it to high blood pressure or diabetes.

*We have to emphasize this to the people, that HIV and AIDS is just the same as diabetes. Why should we hide it? We need to emphasize that to the people…If the community can know that HIV/AIDS is just like any other chronic disease it will be better.* [provider]

While providers reported that HIV is becoming more normalized in health care, reports from community members suggest that some clinic staff are slow to *internalize* these ideas or did not have the vocabulary or training to talk about HIV stigma in a more nuanced way. Community members’ reports implied that providers were still perpetuating stigma and that the providers’ changing narrative about HIV as a normalized health condition had not yet translated into community trust of the health care system. This was also illustrated by the local stigma reduction messages related by some key informants, which focused not only on acceptance, avoiding blame and comparing HIV to diabetes, but also included language around HIV being contracted not just from sex but from helping individuals in car crashes, grannies helping their HIV-positive children without knowing their status, and malicious people sticking others with infected needles – implying that victims of HIV can be ‘innocently’ infected or infected without sexual contact or drug use. Such narratives serve to maintain stigma for ‘some’ people living with HIV and harken back to early in the epidemic when those who were innocently infected by blood donations received empathy and compensation while those who contracted HIV sexually were viewed as ‘bringing upon themselves’ by being ‘promiscuous’ and were deported or denied care and treatment in South Africa [11].

Almost all participants acknowledged the general discomfort with HIV/AIDS owing to its association with sex and sexual transmission. When asked why stigma persisted in the community, respondents reported that the roots of HIV stigma stem from and continue to be HIV/AIDS’s association with sexuality: being HIV-positive signifies behaving immorally or having made a ‘mistake’. Many participants directly associated the silence and stigma surrounding HIV to a general discomfort with the topic of sex. Other participants tacitly made the same link by avoiding discussion of sexual transmission altogether, preferring to talk about HIV outside the context of sex (i.e. contracting HIV from helping their children living with HIV). The obvious deep rooted discomfort and cultural taboos with sex and ensuing lack of communication about sex was accentuated by attempts to disengage sex from conversations about HIV.

### Communication problems at home, in bed and at work

There is ample information about HIV and condoms disseminated in the communities, available through clinics and a variety of community channels (NGOs, schools, government or donor campaigns). The presence of information, however, was not consistent with discussion about HIV/AIDS within the community, within partnerships, within families, or even within provider-client relationships. Most respondents said that information is available and is taught in schools and clinics, but HIV as a topic of exploration is rarely *discussed*. Instead participants reported message fatigue in the form of “*information overload”* and *“suffering from burn out.”* One key informant stated:

*“There was a time when AIDS was over-emphasized. You’d have health officials coming to meetings, talking about HIV and AIDS, and the people would attend meetings, but I think the people felt bombarded with HIV and AIDS.”* [key informant]

The current paradigm around health communication was presented by both community members and providers as extremely didactic and sterile, not creating space for dialogue to help challenge the barriers to HIV prevention and care, particularly for youth. Providers seemed to lack tools to approach HIV outside of a didactic framework and had difficulty with discussions around sex in general. Providers lamented that there were major challenges in how to present information in a palatable way to community members:

*“You go to the health talks, maybe we go and demonstrate condoms. You know? It’s like you’re insulting the men. It is very tough. Very tough.”* [provider]

This lack of discussion and dialogue about HIV and most specifically the lack of communication about sexual transmission was pervasive across participant interviews and was reported in every kind of relationship. Informants reported that families, partners and the community at large, did not talk openly about sex, sexuality, condoms or sexual abuse and therefore any discussion of HIV/AIDS was limited to information provision about where to test and condom use. Participants recognized the importance of discussion around sex theoretically, but had challenges to implement these discussions at home. A lack of communication between parents and children and within intimate relationships was commonly reported, sometimes out of fear of conflict and violence.

*“And of course, parents generally, husbands, wives, they must talk to their children about sex. It’s still taboo even today, as I cannot talk about such things with my children. I’ve been brought up in such a way that I don’t discuss relationships with my children.”* [key informant]

*“We blacks are scared of our men and afraid of talking about our sex life.”* [female community member]

Participants also reported that it was hard to talk about sex “in a deep way” because people felt that sex must “just happen” without a discussion. However, some participants said that couples could and did talk about sex although casual partners are less likely to communicate:

*“… it’s those that love seriously that discuss sex but those that find each other on the streets don’t.”* [community member, female]

There were also many reports concerning a pervasive lack of communication between generations, highlighting what many saw as antagonistic relationships between adults and the youth in both sites. Informants reported “*distorted parent/child relationships”* [key informant] and several adult informants complained about youth becoming unruly and no longer respecting elders because of “*having too many rights*.” Adults reported that youth *“don’t listen”* while youth reported being “*afraid to talk with our parents*” about issues that affect them and that they had no one to talk to when facing relationship hardships. The conflicted inter-generational relationships prevent important conversations between generations. There was a clear need stated from the community for the sensitization of health care workers to working with youth in addition to people living with HIV or other marginalized groups.

### Responding to the Epidemic

A key objective in conducting the situational analysis was to document the current picture of HIV programs and shape ensuing comprehensive programming to address the social barriers to prevention and care and build on programming already in place. Ideally, the programmatic responses would address each of the above named social factors, which facilitate the transmission of HIV and deter prevention, at multiple levels of response. Table 3 outlines current HIV prevention at the different levels of response in addressing the barriers to HIV prevention, testing and treatment that were identified during the fieldwork. We found evidence of top down (national) programs that are mitigating the social risk factors. For example, at the policy level, national programs have facilitated access to testing and treatment, including the world’s largest ARV program. ART eligibility has expanded in the past two years to include anyone with a CD4 count of 350 cells/µL or less, all HIV-positive women who are pregnant or breastfeeding and anyone with WHO clinical stage 3 or 4. As a result, respondents have reported that the increased availability of treatment has combatted HIV fatalism, attributing positive changes in the community specifically to the introduction of ART; the notion that HIV is a ‘death sentence’ has lost traction. One volunteer at an NGO that distributes ARVs recounted a community member’s words:

**Table 3:** Current responses at multiple levels of intervention to address the social contextual factors impacting HIV prevention.

	*HIV Prevalence* *and Fatalism*	*Gender Norms* *and Economics*	*Stigma*	*Communication*
**Government/policy**	Scale up and decentralizationof ART initiation and careand TB and HIV integration;intensive HIV testingcampaigns; primary healthre-engineering to increaseprovision of home,community-based and schoolhealth services – evidencethat this ***counteracts*** ***fatalism***.	RSA’s constitutionis areference for gender equity(though courts donotalways prosecute/followthrough) - potential toimprove genderequity andHIV prevention,but noevidence the community hasinternalized gender equity.*“It’s a long battle to win* *women seeing they have the* *same rights as men.”* [keyinformant]	Anti-discriminationlaws inplace – potential toreduce stigma inhiring/HR practices[76]–[78]. No evidencethis has “trickleddown” intocommunity experience.	Educational campaigns aregovernmentsupported, but generallydidactic in natureand narrowly focused oncondom use andpartner reduction – couldhave improvedfocus on dialogue forchange.
**Clinic** **environment**	Widespread implementationof HCT campaigns andtreatment initiation; verylittle work being done aroundsystematizing efforts toengage and retain patients incare.Providers’ attitudes toepidemic mixed with resignation and hope.*While we’re supposed to be* *[incorporating] families into* *the [treatment retention]* *approach, it’s still led by* *healthcare providers. As* *such, I think we are taking* *the power from the family* *and giving it to the* *healthcare providers. If you* *take power from somebody,* *it will show–they lose* *ownership.* [key informant].	Could improvedialogueabout gender andgenderequity in clinics,where wefound no evidencethat thisdialogue takesplace;victim empowerment unitsare operational, butlimitedin scope. Recognition ofthe obvious genderimbalance accessing clinics– men seek testingand caremore infrequently.	Clinics are oftenstigmatized asbeing associatedwith HIV.Healthcare providersreported notdisclosing theirown statustotheir managers because offearand stigma. Mixedmessagingabout HIV beingnormalizedas a chronic disease butlanguage still infused withstigma. Lack of sensitivitywhile working with keypopulations, such as youthand sex workers*If we as professionals* *also* *couldn’t even dare to-, are* *afraid to [test for HIV], then* *what about the people that* *are not…It really is difficult*[key informant].	Didactic HIV-relatededucation offered andrequired “adherence training” with a focus onpatient “self-control.” Oftena languageof blame – leaves limitedroom forsocial understanding ofdisease or client-centeredcare.
**Local Organizations** **(Churches, NGOs)**	A handful of NGOs provideART, HCT, and support tovery ill – broadens access totreatment and testing (men,farms, very sick, etc.).Some local organizations(mostly churches) werereported to discourage useof condoms or ART –reducing uptake ofprevention/treatment.	One domesticviolencecenter in the area;otherwise very sparse workaround gender andgenderequity.	Some supportgroupsexistedand weredescribed asbeneficialin helpingindividualsdiscloseto family andpartners. Onlyone example ofcommunity-wide stigmareduction workatsites.“*It kills you if* *you keep it in* *your* *heart. It will hurt you. It’s* *better to share these things* *together*.” [communitymember]	Few National NGOs withprogressive campaigns werepresent in the area. LocalNGOs worked in schools andpartnered with clinics,although some reported thatparents didn’t want them inthe schools ‘promoting sex.’It was unclear if NGO staffwere able to stimulatedialogue versus usingtraditional, didactic models.
**Community-based** **response**	No apparent cohesionaround HIV as a communityissue – served tomaintainstatus quo aroundcomplacency regarding HIV.*“One will say they are* *concerned although they* *don’t accept that it may* *happen to them but they* *know… but based on the* *behavior, like seeing a* *lot of young ones being* *pregnant, you ask* *yourself…do they* *really care? They* *know but they seem* *not to care.”* [keyinformant]	Community remains silentaround women’seconomic dependencyand transactional sex andgender-based violence;tacit acknowledgementand little response.Double standards andlanguage ofwomen’s promiscuity –keeps women isolated and ill-informed.	Very few people disclose theirstatus; most fearspeaking outagainst stigma atthecommunity level– createssignificant barriers toprevention behaviors, andaccessing testing and care.	Strong taboos around talking aboutsex – creates hostile environmentto talk about *safe* sex.“*Parents discourage talking about* *safe sex because they think it will* *encourage children to have sex*”[provider]

*“She said to me, “Ever since you opened, we are no longer having too many funerals. We came to tell you, thank you. We are so grateful.” And, you know what, I think she is right… after all the awareness and the rolling out of ARVs… it is true.”* [key informant]

At a policy level, the national government has also adopted anti-discrimination laws and gender-equitable language, policies that should serve as a reference for anti-discrimination and gender equity.

We found little evidence that these national policies are effectively reaching the rural communities to change the enactment of HIV stigma or gender norms. Respondents reported a lack of interest and little faith in pursuing cases of domestic violence, although this was partially attributed to lack of responsiveness by police and local criminal justice systems.

In addition, the national laws may not be implemented in practice as they are established in theory and national policies may be differentially implemented at the provincial and district level; we did not hear reports from participants living with HIV that they felt protected in terms of a legal right to equal treatment, though they did acknowledge their right to health care. Along the same lines, the laws against gender-based violence in the constitution are strong, but not consistently applied and compliance is certainly less than perfect across the judicial system [28], [29].

At the level of the health delivery system, treatment availability has also served to combat HIV fatalism, though it was clear that the intention of national policies to ensure a clinic environment free of stigma and discrimination are unlikely to be consistently implemented without ensuring that health personnel are trained to offer care without judgment or stigmatization. Providers did not report systematically receiving any training to combat stigma or to work with stigmatized groups, such as men who have sex with men (MSM) or sex workers. Instead, many community members reported feeling stigmatized and judged by providers and were generally distrustful of clinics. At the same time that clinicians often feel overburdened with the patient load and reported a sense of low morale, some providers also lamented not gaining more skills to communicate with their clients – particularly with young people. At the clinic level, programming to address social barriers to care is scarce.

Finally, we sought out evidence of *community-based* responses to HIV, responses generated from the community or community organizations and often from the ground-up, which can address social risk factors to HIV. Despite the ubiquitous presence of HIV in the study communities, there was not a home-grown prevention response to the epidemic. While one community housed several national NGO offices, community-based efforts extended only to home based care volunteers, some of whom received government stipends, and a handful of clinic-based support groups. Though there was ample recognition of social barriers to HIV prevention and care, communities were not engaged in their own prevention or treatment campaigns. There were also no examples of community responses to domestic violence in our data; there was one government-funded domestic violence shelter, but when our group visited, they had no clients.

## Discussion

Comprehensive prevention programs can only be successfully designed and implemented if proper attention is given to identifying not only the characteristics of the local epidemic and the health sector and programs in place, but also the factors in the social environment that are shaping health and behavior, specifically those that are amenable to change [12], [30]. We found that HIV fatalism, traditional gender norms, HIV-related stigma, and lack of communication around sex and HIV prevention contribute to the on-going HIV epidemic in two rural areas of North West province, South Africa. The social barriers to HIV prevention that surfaced as most prominent in our data parallel previous qualitative research in South Africa [31]–[33]. We also noted that there are few current responses to the epidemic in the area that address these social factors – instead there is neither community response or action in the clinics to expand HIV prevention and care efforts nor efforts to address issues such as gender equity and HIV stigma, though government policies and programs are in place to serve as references for forward progress in these areas. Ideally national programming aligns with and bolsters community responses to the epidemic, creating a more effective prevention synergy [34]. In the case of the rural Northwest Province, the community response is mostly absent and the national programming, while responsive to collective voices in urban South Africa, seems distant and not always well understood or consistently implemented.

The presence of extensive HIV fatalism has been previously documented in South Africa, where qualitative research has found that youth expect to contract HIV [35] and that fatalism and ‘bravado’ facilitate sexual risk behavior [36]. The consequences of the normalization of HIV in South Africa and the mental health effects of living in communities faced with such dramatic rates of HIV and HIV-related deaths are poorly understood [37]. The roots of HIV fatalism, are not surprising, however, from either an epidemiologic or a historical perspective, given South Africa’s HIV history – from the apartheid government’s painting HIV as a black disease; barring Blacks and MSM from donating blood, building a campaign focused on fear and stigma, to the ANC’s history of HIV denialism [11]. The current government of President Jacob Zuma has expended significant efforts on damage control, beginning with progressive national policies and expanded coverage for HIV care. However, this more progressive vision has not fully reached the distant rural communities where this situational analysis was conducted and where residual denialism and community trauma has left widespread resignation to HIV. Despite universal ART access, many people report barriers to ART care [38], although the expansion of treatment and care represents a beginning to breaking this cycle of resignation.

We found that adherence to traditional gender norms in the area place women and men at risk of HIV acquisition and work as an obstacle to obtaining and adhering to HIV care. We documented multiple reports of violence against women and tacit silence; adherence to traditional ideas of masculinity that encourage multiple sexual partnerships; and blaming of women for the HIV epidemic at the same time that women’s economic dependency on men forces unequal power dynamics. Significant cross-sectional and qualitative research in South Africa has been conducted on gender norms that define men and women’s rights, socially accepted behavior, decision-making power, and relationship to each other and HIV – noting that inequalities between men and women has fueled the HIV epidemic [39]. Adherence to traditional norms of masculinity has been associated with increased intimate partner violence, multiple sexual partnerships, increased likelihood of STI/HIV infection, and a reduction in health seeking services [23], [40]–[43]. Other research has found that without adequate engagement, men feel left behind or backlash against women’s new rights [44]. Indeed, South Africa has high rates of violence against women, which both exacerbates and reflects this imbalance in gender norms [45]. We also noted a significant tension created by normative expectations around women’s sexuality and economic demands of exchanging sex for food or survival. Informal transactional sex fueled by economic dependency is well documented throughout South Africa [46]–[48], where women often stay in potentially abusive relationships when economically dependent [49]. While programs to change or mitigate gender norms for HIV prevention have some success [26], gender norms programming has been mostly limited to NGO-based, short-term group-based programs, whereas strides will require efforts across multiple levels of programming, including structural interventions and governmental efforts [26], [50].

Our research documented that while communities are in transition regarding HIV stigma, it remains a significant barrier to condom use, HIV testing and treatment. Stigma stymied open dialogue about HIV and created an environment of silence and fear, echoing previous research in communities across South Africa [51], [52]. Similar to our findings, investigators have found that the introduction of ARTs in rural South Africa has shifted the narrative around HIV stigma, but that stigma still persists [53]. Stigma has been found in health care settings and is associated with low access to care and poorer physical and mental health outcomes [54], [55]. While providers in our sample seem to have received some instruction around how to frame discussions about HIV stigma as well as principles of quality care (e.g., maintaining patient confidentiality), it appears that the reality of discriminatory messaging is still very present. Providers often reported that HIV is ‘like any other chronic disease,’ but often the same providers’ language of disapproval around sexual behavior would imply otherwise. As with changing gender norms, prevention programming to combat stigma will require reinforcing messages at multiple levels – in policies, in clinics, in the communities [55], [56] – which is not yet in evidence in this rural area.

Finally, open dialogue and frank communication around sex and HIV appeared to be rare occurrences in the experience of our participants, though participants reported receiving ‘instruction’ about HIV prevention. The discomfort communicating about sex that was evident in our data at the individual, interpersonal, and community level, serves to stymie HIV prevention efforts. In South Africa, antagonistic parental relationships and a lack of open dialogue have been linked to increased HIV risk [57], [58]. Some have hypothesized that the long-standing denialism around HIV/AIDS espoused by country leaders has perpetuated parents’ refusal to accept or talk openly about sex [58]. This lack of intergenerational discussion is unfortunate, particularly because research among US adolescents has demonstrated that openly communicating with parents about HIV was associated with greater competence in talking about HIV with sexual partners [59]. One approach to improve communication around sex is Couples HIV Counseling and Testing (CHCT), which has been demonstrated to be an effective intervention for sexual risk reduction [60] and is promoted by the South African government. Qualitative studies of couples from South Africa and Uganda have reported that couples value relationship-based factors such as trust and commitment, and express interest in being able to communicate with each other about difficult topics such as HIV and its impact on their relationships [61], [62]. However, CHCT uptake is quite low [63], and while stated reasons for lack of uptake include fear of disclosure and violence, we also hypothesize that the lack of communication around sex is so intrinsic to the experience of couples in South Africa that even communication about accessing CHCT becomes daunting. There is an urgent need for community-based programs to improve communication skills for couples, including both general skills as well as for sex and HIV, and for programs that generate dialogue around gender, relationships, and open communication for couples and communities.

Our data also implies that there is a significant need for constructive communication and a shift in how communities are receiving information about safe sex, HIV prevention, and treatment. Unlike didactic models, consciousness-raising models that promote community empowerment through dialogue have the capacity to unleash communication and promote deeper understanding and thus thoughtful responses to health conditions and the social context that fuels them [31], [32], [64]; this model has had success in an African context increasing gender equitable ideals, self-efficacy and agency [65]. Some programs that encourage families to communicate about sex have proven successful [66]; however expansion of programs such as these might hit road blocks from churches and other traditional stakeholders, particularly in rural areas.

### Implications for Programming

Research has found that community-based programming has the potential for significant impacts on the HIV epidemic but only if programming fits the actual needs of the community as opposed to a “one-size fits all” approach [34]. The situational analysis described herein served to inform a large-scale comprehensive HIV prevention program, currently underway, that aims to address social barriers to prevention and care. This assessment provided a snapshot of the social context, revealed targeted themes for prevention programming, and afforded an overview of current programming and community resources. While a lengthy description of the program is not the focus of the current paper, we describe program components below, including implementation of four strategies, which all incorporated the themes identified in our approach.

Thematic *content* based on our findings in the situational analysis, was infused into four comprehensive prevention *strategies* that include community-based testing, support groups for HIV positive people, health systems strengthening, and community engagement. We are implementing community-based testing programs; Community-based HIV, STI and TB testing facilitates entry into services in areas where access to care is particularly tenuous and where a lack of community trust in the health care workers, mostly due to stigmatizing attitudes, keeps people out of the clinic. The community-based services are designed to provide HCT incorporated into a general health promotion strategy, including blood pressure and diabetes monitoring, to reinforce a holistic approach to health in a non-judgmental and open space where education and dialogue around programmatic themes can occur. The need for improved communication regarding sex and HIV prevention in intimate relationships led to offering CHCT as a component of the community-based HIV testing program, where the counseling teams underwent an intensive course to encourage open discussion between partners that accounts for HIV test results of both partners and the ensuing serostatus of the couple (HIV concordant, serodiscordant, etc.) with regard to potential challenges regarding sexual risk for HIV and fertility. Unfortunately, uptake of CHCT in the first quarter of the program was extremely low, reflecting the experience of other programs offering CHCT in South Africa and emblematic of the greater societal discomfort discussing sex, even in intimate relationships, which certainly contributes to HIV transmission. The program is currently re-visiting approaches to encourage dialogue and communication around sex.

The second strategy includes support groups for people living with HIV/AIDS (PLHIV) to encourage treatment adherence and to create an environment conducive for building social networks that can reduce stigma and engender support. The support group content was designed to respond to the themes found in the situational analysis, including fostering dialogue around gender norms and stigma, communication skills about sex and HIV, and coping with being HIV-positive living positively and healthily (including prevention and adherence). We also have devised a health systems strengthening component, which aims to address barriers to quality care within the system, including trainings for providers and staff on the assessment themes: stigma, confidentiality, and working with stigmatized groups; gender norms and skills for addressing gender based violence; and general communication skills around sex. Finally, community engagement is a significant component of the project–supporting efforts to cultivate community responses to the epidemic, reduce stigma, challenge harmful gender norms and combat HIV fatalism. Project staff design, organize and facilitate community events that are meant to bring skills to the community–such as gardening and entrepreneurship – as well as promote dialogue and discussion around issues of gender, stigma, sex and HIV. Stakeholders groups and community working groups have been established to support the program goals and explore means to drive community and local policy responses to the epidemic. An evaluation is currently underway to determine whether the approach taken and activities being conducted both impact the targeted issues encapsulated by the themes and improve access to testing, prevention, care, ultimately reducing the burden of disease in project sites.

### Strengths and Limitations

The situational analysis method was flexible and iterative in nature – allowing for developing and then validating hypotheses during stakeholder meetings and fieldwork – and was done rapidly, so that findings could influence program design without the long delays of traditional formative work [67]. This approach also emphasizes ample community involvement, which increases the likelihood of program success by utilizing community knowledge and establishing early partnerships [9]. The situational analysis was a powerful tool to simultaneously facilitate community entry and buy-in, with the success of the situational analysis based on engaging stakeholders and documenting the social context and means to address underlying processes that place people at risk for HIV. At the same time, an inherent drawback is that the speed of data collection can come at the expense of depth. For example, other potentially vulnerable sub-communities (LGBT, white, Indian) were not explored in depth because of a lack of time and the relatively small number of people in each of those groups. However, it’s not clear that our program would have been able to reach that audience with its limited resources. Finally, while we gathered available epidemiologic data to better understand the state of the epidemic, we did not capture detailed quantitative data during the fieldwork, which is included in some rapid assessment approaches [68], and can help to understand local trends at the sub-district level to assist with targeting programs.

### Conclusions

There is currently broad demand for HIV prevention and care interventions that address social and structural risk factors, [69]–[71] and yet there have been few programs to date in Southern Africa with successful examples of implementing comprehensive prevention approaches that address social barriers to prevention and care [40] [42] [72], [73]. Because multiple and complementary approaches are needed to engage communities, providers, and local leaders simultaneously, exploring community, provider, and stakeholder perspectives is key to building comprehensive programs, as is engaging these groups in developing a response. We believe that beginning the planning process with a situational analysis is a crucial step to design and implement comprehensive programming that addresses social barriers to care and engages key groups. In our project sites, prior to the situational analysis, we found little evidence that discussion had occurred regarding the social structures that inhibit progress in stemming the HIV epidemic. With the implementation of the type of tailored programming made possible by this approach, we hope to lessen the impact of social barriers to prevention and care, and, ultimately improve the quality of HIV-related services that are available– thus decreasing the burden of HIV experienced in these communities.

## References

[1] Shisana RT, Simbayi LC, Zuma K, Jooste S, Zungu N, et al.. (2014) South African National HIV Prevalence, Incidence and Behaviour Survey, 2012. Cape Town: HSRC Press. 10.2989/16085906.2016.1153491 27002359

[2] UNAIDS (2012) UNAIDS Country Profile: South Africa.

[3] World Health Organization (2013) Global Tuberculosis Report.

[4] South African National Department of Health (2012) Clinical Guidelines for the Management of HIV&AIDS in Adults and Adolescents.

[5] UNAIDS (2010) Global Report: UNAIDS Report on the Global AIDS Epidemic.

[6] Johnson LF (2012) Access to antiretroviral treatment in South Africa, 2004–2011.

[7] PEPFAR (2011) Guidance for the prevention of sexually transmitted HIV Infections.

[8] Coates TJ, Richter Linda, Caceres Carlos (2008) Behavioural strategies to reduce HIV transmission: how to make them work better. The Lancet 372: 669–684. 10.1016/S0140-6736(08)60886-7 PMC2702246 18687459

[9] Kevany S, Khumalo-Sakutukwa G, Murima O, Chingono A, Modiba P, et al (2012) Health diplomacy and Adapting global health interventions to local needs: findings from project accept (HPTN 043), a community-based intervention to reduce HIV incidence in populations at risk in Sub-Saharan Africa and Thailand. BMC Public Health 12: 459. 22716131 10.1186/1471-2458-12-459 PMC3552975

[10] Auerbach JD, Parkhurst JO, Caceres CF (2011) Addressing social drivers of HIV/AIDS for the long-term response: conceptual and methodological considerations. Glob Public Health 6: 11. 10.1080/17441692.2011.594451 21745027

[11] Karim SSA, Churchyard GJ, Karim QA, Lawn SD (2010) HIV infection and tuberculosis in South Africa: an urgent need to escalate the public health response. The Lancet 374: 921–933. 10.1016/S0140-6736(09)60916-8 PMC2803032 19709731

[12] Campbell C, Nair Y, Maimane S (2007) Building contexts that support effective community responses to HIV/AIDS: a South African case study. Am J Community Psychol 39: 347–363. 17447133 10.1007/s10464-007-9116-1

[13] Manderson L, Aaby P (1992) An epidemic in the field? Rapid assessment procedures and health research. Social Science & Medicine 35: 839–850. 1411684 10.1016/0277-9536(92)90098-b

[14] World Health Organization (2005) Strategic approach to improving quality of care in reproductive health services. Geneva: Department of Reproductive Health and Research.

[15] Lippman SA, Kerrigan D, Chinaglia M, Diaz J (2007) Chaos, co-existence and the potential for collective action: HIV-related vulnerability in Brazil’s international borders. Social Science and Medicine 64: 2464–2475. 17434653 10.1016/j.socscimed.2007.02.048

[16] Spicehandler J, Simmons R (1994) Expanding family planning options. Contraceptive introduction reconsidered: A review and conceptual framework. Geneva: WHO Special Program of Research, Development and Research Training in Human Reproduction.

[17] World Health Organization (2002) Making decisions about contraceptive introduction: A guide for conducting assessments to broaden contraceptive choice and improve quality of care. Geneva: WHO Department of Reproductive Health and Research.

[18] Treves-Kagan S, Naidoo E, Gilvydis J, Barnhart S, Lippman SA (In draft ) A rapid assessment methodology to inform comprehensive HIV prevention programming, rural North West Province, South Africa.

[19] National Department of Health (2012) The National Antenatal Sentinel HIV and Syphilis Prevalence Survey, South Africa, 2011. Pretoria.

[20] Ulin P, Robinson E, Tolley E (2005) Qualitative Methods in Public Health: A Field Guide for Applied Research. San Francisco: Jossey-Bass.

[21] Miles M, Huberman M (1994) Qualitative Data Analysis: An Expanded Sourcebook. London: Sage Publications.

[22] Jewkes R, Morrell R (2010) Gender and sexuality: emerging perspectives from the heterosexual epidemic in South Africa and implications for HIV risk and prevention. Journal of the International AIDS Society 13: 6. 20181124 10.1186/1758-2652-13-6 PMC2828994

[23] Jewkes R, Sikweyiya Y, Morrell R, Dunkle K (2011) The Relationship between Intimate Partner Violence, Rape and HIV amongst South African Men: A Cross-Sectional Study. PLoS ONE 6(9): e24256 doi:10.1371/journal.pone.0024256 21935392 10.1371/journal.pone.0024256 PMC3173408

[24] Kalichman SC, Simbayi, L C, Cain D, Cherry C, Henda N (2007) Cloete A (2007) Sexual assault, sexual risks and gender attitudes in a community sample of South African men. AIDS Care 19: 20–27. 17129854 10.1080/09540120600984003

[25] Pulerwitz J, Barker G (2004) Promoting Health Relationships and HIV/STI Prevention for Young Men: Positive Findings from an Intervention Study in Brazil. Washington, DC: Population Council.

[26] Dworkin SL, Treves-Kagan S, Lippman SA (2013) Gender-Transformative Interventions to Reduce HIV Risks and Violence with Heterosexually-Active Men: A Review of the Global Evidence. AIDS and Behavior 17: 2845–2863. 23934267 10.1007/s10461-013-0565-2

[27] South African National AIDS Council (2010) The National HIV Counselling and Testing Campaign Strategy South African Department of Health.

[28] Peacock D, Stemple L, Sawires S, Coates TJ (2009) Men, HIV/AIDS, and human rights. J Acquir Immune Defic Syndr 1. 10.1097/QAI.0b013e3181aafd8a PMC2853958 19553779

[29] Stemple L (2008) Health and human rights in today’s fight against HIV/AIDS. AIDS 22. 10.1097/01.aids.0000327443.43785.a1 PMC3356156 18641463

[30] Merson M, Padian N, Coates TJ, Gupta GR, Bertozzi SM, et al (2008) Combination HIV Prevention. Lancet 372: 1805–1806. 10.1016/S0140-6736(08)61752-3 19027478

[31] Lippman SA, Maman S, MacPhail C, Twine R, Peacock D, et al (2013) Conceptualizing community mobilization for HIV prevention: implications for HIV prevention programming in the African context. PLOS ONE 8(10): e78208 doi:10.1371/journal.pone.0078208 24147121 10.1371/journal.pone.0078208 PMC3795620

[32] Campbell C (2003) ‘Letting them Die’ Why HIV/AIDS Prevention Programmes Fail. Bloomington: Indiana University Press.

[33] Campbell C, MacPhail C (2002) Peer education, gender and the development of critical consciousness: participatory HIV prevention by South African youth. Social Science and Medicine 55: 331–345. 12144146 10.1016/s0277-9536(01)00289-1

[34] Rodriguez-Garcia R, Wilson D, York N, Low C, N’Jie N, et al (2013) Evaluation of the community response to HIV and AIDS: learning from a portfolio approach. AIDS Care 25: 764395. 10.1080/09540121.2013.764395 PMC4003575 23745633

[35] Leclerc-Madlala S (1997) Infect one, infect all: Zulu youth response to the aids epidemic in South Africa. Medical Anthropology 17: 363–380. 9241993 10.1080/01459740.1997.9966146

[36] Campbell C, Foulis CA, Maimane S, Sibiya Z (2005) The impact of social environments on the effectiveness of youth HIV prevention: A South African case study. AIDS Care 17: 471–478. 16036233 10.1080/09540120412331319705

[37] Jewkes R (2006) Beyond stigma: social responses to HIV in South Africa. The Lancet 368: 430–431. 10.1016/S0140-6736(06)69130-7 16890812

[38] Govindasamy D, Ford N, Kranzer K (2012) Risk factors, barriers and facilitators for linkage to antiretroviral therapy care: a systematic review. AIDS 26: 2059–2067. 22781227 10.1097/QAD.0b013e3283578b9b

[39] Mantell JE, Needham SL, Smit JA, Hoffman S, Cebekhulu Q, et al (2009) Gender norms in South Africa: implications for HIV and pregnancy prevention among African and Indian women students at a South African tertiary institution. Cult Health Sex 11: 139–157. 19247859 10.1080/13691050802521155 PMC2782559

[40] Jewkes R, Nduna M, Levin J, Jama N, Dunkle K, et al.. (2008) Impact of Stepping Stones on incidence of HIV and HSV-2 and sexual behaviour in rural South Africa: cluster randomised controlled trial. BMJ 337. 10.1136/bmj.a506 PMC2505093 18687720

[41] Jewkes RK, Dunkle K, Nduna M, Shai N (2010) Intimate partner violence, relationship power inequity, and incidence of HIV infection in young women in South Africa: a cohort study. Lancet 376: 41–48. 20557928 10.1016/S0140-6736(10)60548-X

[42] Kalichman S, Simbayi L, Cloete A, Clayford M, Arnolds W, et al (2009) Integrated Gender-Based Violence and HIV Risk Reduction Intervention for South African Men: Results of a Quasi-Experimental Field Trial. Prevention Science 10: 260–269. 19353267 10.1007/s11121-009-0129-x PMC3015088

[43] Courtenay WH (2000) Constructions of masculinity and their influence on men’s well-being: a theory of gender and health. Social Science and Medicine 50: 1385–1401. 10741575 10.1016/s0277-9536(99)00390-1

[44] Dworkin SL, Dunbar MS, Krishnan S, Hatcher AM, Sawires S (2011) Uncovering Tensions and Capitalizing on Synergies in HIV/AIDS and Antiviolence Programs. American Journal of Public Health 101: 995–1003. 21164091 10.2105/AJPH.2009.191106 PMC3093292

[45] Mosavel M, Ahmed R, Simon C (2012) Perceptions of gender-based violence among South African youth: implications for health promotion interventions. Health Promotion International 27: 323–330. 21733916 10.1093/heapro/dar041 PMC3411193

[46] Dunkle KL, Jewkes RK, Brown HC, Gray GE, McIntryre JA, et al (2004) Transactional sex among women in Soweto, South Africa: prevalence, risk factors and association with HIV infection. Social science & medicine 59: 1581–1592. 15279917 10.1016/j.socscimed.2004.02.003

[47] Dunkle KL, Jewkes R, Nduna M, Jama N, Levin J, et al (2007) Transactional sex with casual and main partners among young South African men in the rural Eastern Cape: prevalence, predictors, and associations with gender-based violence. Soc Sci Med 65: 1235–1248. 17560702 10.1016/j.socscimed.2007.04.029 PMC2709788

[48] Kaufman CE, Stavrou SE (2004) ‘Bus fare please’: the economics of sex and gifts among young people in urban South Africa. Culture, Health & Sexuality 6: 377–391.

[49] Pitpitan EV, Kalichman SC, Cain D, Eaton LA, Carey KB, et al (2012) Condom Negotiation, HIV Testing, and HIV Risks among Women from Alcohol Serving Venues in Cape Town, South Africa. PLoS ONE 7: e45631. 23056211 10.1371/journal.pone.0045631 PMC3462794

[50] Greig A, Peacock D, Jewkes R, Msimang S (2008) Gender and AIDS: time to act. AIDS 22: 18. 10.1097/01.aids.0000327435.28538.18 PMC3356155 18641466

[51] Kalichman SC, Simbayi LC (2003) HIV testing attitudes, AIDS stigma, and voluntary HIV counselling and testing in a black township in Cape Town, South Africa. Sexually Transmitted Infections 79: 442–447. 14663117 10.1136/sti.79.6.442 PMC1744787

[52] Skinner D, Mfecane S (2004) Stigma, discrimination and the implications for people living with HIV/AIDS in South Africa. SAHARA-J: Journal of Social Aspects of HIV/AIDS 1: 157–164. 17601003 10.1080/17290376.2004.9724838

[53] Zuch M, Lurie M (2012) ‘A virus and nothing else’: the effect of ART on HIV-related stigma in rural South Africa. AIDS Behav 16: 564–570. 22080386 10.1007/s10461-011-0089-6

[54] Kinsler JJ, Wong MD, Sayles JN, Davis C, Cunningham WE (2007) The effect of perceived stigma from a health care provider on access to care among a low-income HIV-positive population. AIDS Patient Care STDS 21: 584–592. 17711383 10.1089/apc.2006.0202

[55] Nyblade L, Stangl A, Weiss E, Ashburn K (2009) Combating HIV stigma in health care settings: what works? Journal of the International AIDS Society 12: 15. 19660113 10.1186/1758-2652-12-15 PMC2731724

[56] Mahajan AP, Sayles JN, Patel VA, Remien RH, Sawires SR, et al (2008) Stigma in the HIV/AIDS epidemic: a review of the literature and recommendations for the way forward. AIDS 22: 62. 10.1097/01.aids.0000327438.13291.62 PMC2835402 18641472

[57] MacPhail C, Campbell C (2001) ‘I think condoms are good but, aai, I hate those things’:: condom use among adolescents and young people in a Southern African township. Social Science and Medicine 52: 1613–1627. 11327136 10.1016/s0277-9536(00)00272-0

[58] Lambert H, Wood K (2005) A comparative analysis of communication about sex, health and sexual health in India and South Africa: Implications for HIV prevention. Cult Health Sex 7: 527–541. 16864220 10.1080/13691050500245818

[59] Shoop DM, Davidson PM (1994) AIDS and adolescents: the relation of parent and partner communication to adolescent condom use. Journal of Adolescence 17: 137–148.

[60] Rosenberg NE, Pettifor AE, De Bruyn G, Westreich D, Delany-Moretlwe S, et al (2013) HIV testing and counseling leads to immediate consistent condom use among South African stable HIV-discordant couples. J Acquir Immune Defic Syndr 62: 226–233. 23117500 10.1097/QAI.0b013e31827971ca PMC3548982

[61] Darbes LA, van Rooyen H, Hosegood V, Ngubane T, Johnson MO, et al (2014) Uthando Lwethu (‘our love’): a protocol for a couples-based intervention to increase testing for HIV: a randomized controlled trial in rural KwaZulu-Natal, South Africa. Trials 15: 64. 24552199 10.1186/1745-6215-15-64 PMC3936910

[62] Ware NC, Wyatt MA, Haberer JE, Baeten JM, Kintu A, et al (2012) What’s love got to do with it? Explaining adherence to oral antiretroviral pre-exposure prophylaxis for HIV-serodiscordant couples. J Acquir Immune Defic Syndr 59: 463–468. 22267018 10.1097/QAI.0b013e31824a060b PMC3826169

[63] Medley A, Baggaley R, Bachanas P, Cohen M, Shaffer N, et al (2013) Maximizing the impact of HIV prevention efforts: interventions for couples. AIDS Care 25: 1569–1580. 23656251 10.1080/09540121.2013.793269 PMC4664148

[64] Freire P (1970) Pedagogy of the oppressed. New York: Herder and Herder.

[65] Underwood C, Brown J, Sherard D, Tushabe B, Abdur-Rahman A (2011) Reconstructing Gender Norms Through Ritual Communication: A Study of African Transformation. Journal of Communication 61: 197–218.

[66] Vandenhoudt H, Miller KS, Ochura J, Wyckoff SC, Obong’o CO, et al (2010) Evaluation of a U.S. evidence-based parenting intervention in rural Western Kenya: from parents matter! To families matter! AIDS Educ Prev. 22: 328–343. 10.1521/aeap.2010.22.4.328 20707693

[67] Fitch C, Rhodes T, Stimson GV (2000) Origins of an epidemic: the methodological and political emergence of rapid assessment. International Journal of Drug Policy 11: 63–82. 10699545 10.1016/s0955-3959(99)00056-0

[68] Trotter RT, Needle RH, Goosby E, Bates C, Singer M (2001) A Methodological Model for Rapid Assessment, Response, and Evaluation: The RARE Program in Public Health. Field Methods 13: 137–159.

[69] Sumartojo E (2000) Structural factors in HIV prevention: concepts, examples, and implications for research. AIDS 14: S3–S10. 10.1097/00002030-200006001-00002 10981469

[70] Gupta GR, Parkhurst JO, Ogden JA, Aggleton P, Mahal A Structural approaches to HIV prevention. The Lancet 372: 764–775. 10.1016/S0140-6736(08)60887-9 18687460

[71] Parker RG, Easton D, Klein CH (2000) Structural barriers and facilitators in HIV prevention: a review of international research. AIDS 14: S22–S32. 10981471 10.1097/00002030-200006001-00004

[72] Sweat M, Morin S, Celentano D, Mulawa M, Singh B, et al (2011) Community-based intervention to increase HIV testing and case detection in people aged 16–32 years in Tanzania, Zimbabwe, and Thailand (NIMH Project Accept, HPTN 043): a randomised study. Lancet Infect Dis 11: 525–532. 21546309 10.1016/S1473-3099(11)70060-3 PMC3156626

[73] Pettifor A, MacPhail C, Kahn K (2009–2014) Effects of Cash Transfer and Community Mobilization in Young South African Women (1R01MH087118–01/HPTN 068). Mmpulanga, South Africa: NIH/NIMH/HPTN.

[74] StatsSA (2011) Community Survey.

[75] StatsSA (2007) Community Survey - Labor Force.

[76] Fredman S (2012) Comparative study of anti-discrimination and equality laws of the US, Canada, South Africa and India. European Commission.

[77] (1998) Employment Equity Act: Code of Good Practice on HIV and AIDS and the World of Work. Act No. 55 of 1998. South Africa.

[78] (1996) Constitution of the Republic of South Africa. South Africa.

